# Uncovering Barriers to Prenatal Physical Activity and Exercise Among South African Pregnant Women: A Cross-Sectional, Mixed-Method Analysis

**DOI:** 10.3389/fpubh.2022.697386

**Published:** 2022-04-01

**Authors:** Uchenna Benedine Okafor, Daniel Ter Goon

**Affiliations:** ^1^Department of Nursing Science, University of Fort Hare, Alice, South Africa; ^2^Department of Public Health, University of Fort Hare, Alice, South Africa

**Keywords:** physical activity, exercise, barriers, pregnant women, South Africa

## Abstract

**Background:**

The barriers to prenatal physical activity and exercise have been widely reported in the literature, highlighting context-specific challenges. However, generally, research on prenatal physical activity and exercise among pregnant women in South Africa is rare; and particularly concerning the barriers to their prenatal physical activity practice. This study assessed the barriers to physical activity participation among pregnant women in the Eastern Cape, South Africa.

**Methods:**

This was a sequential explanatory mixed method, predominantly quantitative study involving 1,082 pregnant women. A structured self-administered questionnaire on perceived barriers to physical participation was applied to collect quantitative data; while a subset of 15 pregnant women participated in individual in-depth semi-structured interviews to augment quantitative data. Quantitative and qualitative data were analyzed using descriptive statistics and thematic analysis, respectively.

**Results:**

The results of the quantitative analysis presented major barriers: lack of advice on prenatal physical activity and inadequate or conflicting information about prenatal physical activity; tiredness; work commitments; discomfort; lack of time; low energy; non-accessibility to physical activity; lack of financial resources, and safety concerns for the mother and the baby. Qualitatively, the barriers identified relate to four main themes: individual, lack of information, lack of resources, and environmental barriers.

**Conclusion:**

The major barriers cited by the women were tiredness, lack of time, discomfort, and low energy. They also include lack of support, advice and information about prenatal physical activity. The multiple constraining factors responsible for low or non-prenatal physical activity of pregnant women in this setting highlight the need to tailor interventions to address their individual uniquely perceived barriers.

## Introduction

Worldwide, and in the limited studies in South Africa (1–5), prenatal physical activity and exercise reportedly remain low, despite their benefits for the mother's health and that of the unborn child, and even beyond childbirth. Existing literature indicates context-specific limiting factors–social, economic, and cultural ones affecting women's decisions to initiate, participate and continue physical activity and exercise during pregnancy. In addition, the physiological and anatomical changes during pregnancy could constitute barriers to physical activity (6).

The factors hindering physical activity participation during pregnancy are multi-dimensional and geographically specific. The most commonly and frequently cited barriers to physical activity during pregnancy are tiredness, pregnancy symptoms and discomfort (1, 7–9); lack of strength or fatigue (5, 7, 8), lack of time (1, 7, 8, 10), lack of motivation (5, 11, 12), lack of social support (13–15), and concern about the safety of physical activity for the baby and the mother (5, 11, 16–18). In addition, cultural and religious beliefs (19, 20), and children, work, and family responsibilities or commitments (15, 21–23) are also consistently listed as constraining factors to physical activity during pregnancy. Environmentally, limited accessibility to facilities and resources (24, 25), and bad weather conditions (8, 15, 24) are considered deterrents to physical activity. These barriers have a profound influence in restraining physical activity participation, and research is needed to clarify the issues and indicate solutions.

An understanding of the barriers preventing women from being physically active during pregnancy is important to inform future interventions to improve physical activity levels in pregnancy. However, generally, research on prenatal physical activity and exercise among pregnant women in South Africa is rare; and to the best of the author's knowledge, there are very few studies, if any, applying a mixed-method approach to assess the barriers to physical activity and exercise among pregnant women in the Eastern Cape Province. Applying a mixed-methods approach would provide unique, deeper insights or understanding of the barriers to prenatal physical activity by integration of both quantitative and qualitative data, than either approach alone (26–28). Therefore, the objective of this study was to identify and explore the underlying factors restraining pregnant women from physical activity and exercise during pregnancy in Buffalo City Municipality, Eastern Cape, South Africa.

## Materials and Methods

### Study Setting, Participant, and Design

The study was conducted among pregnant women in Buffalo City Municipality in Eastern Cape Province, South Africa. Data was collected from July to October 2019. The details describing the study setting has been described elsewhere (29). A health-facility based sequential explanatory mixed method (quantitative and qualitative) approach was used to identify and explore barriers to prenatal physical activity and exercise through a structured questionnaire and in-depth individual interviews. The qualitative research was used to provide in-depth information on barriers to physical activity during pregnancy which might not have being listed in the quantitative questionnaire, thereby also granting the women an opportunity to express their views without limitation.

First, we applied a random table selection technique to select 12 primary health clinics out of the 72 health facilities in Buffalo City Municipality; next, we approached the women attending antenatal appointments and explained the aim and nature of the study to them; after that we conveniently selected those who were willing and eligible to participate in the study. Hence, the study was conducted at the health clinics, convenient sample was deemed appropriate because of cost and easy accessibility of the participants.

Participants were included in the study if 18 years and above, receiving antenatal care in the selected health clinics, single pregnancy, and could read or understand the IsiXhosa, Afrikaans or English languages. We excluded women with disabilities or reasons to cease exercise at the time of recruitment such as “persistent excessive shortness of breath, dizziness or faintness that does not resolve on rest, regular and painful uterine contractions, vaginal bleeding, severe chest pain, and persistent loss of fluid from vagina indicating rupture of the membranes” (30).

The Sarmah et al. (31) formulae was applied to calculate the sample size, which has been reported in a previous publication (32). We recruited 1,215 participants in the quantitative study; however, due to eligibility concerns, refusal and missing information on medical records, 1,082 were eventually included in the data analysis. Twenty-four pregnant women attending antenatal healthcare appointments in the selected health clinics were purposively selected to participate in the qualitative interviews, that is, two pregnant women per selected primary health clinic who met the inclusion criteria were selected for interview. However, due to interview data saturation, 15 pregnant women comprised the participated in individual in-depth semi-structured interviews during antenatal visits.

### Ethical Considerations

The study protocol was approved by the University of Fort Hare's Health Research Ethics Committee (Ref#2019 = 06 = 009 = OkaforUB). In addition, permission was obtained from the Eastern Cape Department of Health and from all the selected health clinics before data collection. The nature and aim of the study was explained to the participants. They were assured of voluntary participation and could withdraw from the study at any time, without any prejudice or penalty. Women who accepted to participate in the study provided their written informed consent. The confidentiality, anonymity and rights of the participants were strictly adhered to and respected.

### Quantitative Data

Quantitative data were collected in 12 selected health clinics by the primary investigator (BUO) and the research assistants. Interviewer-administered structured closed-ended questionnaire was used to collect quantitative data on socio-demographic (racial affiliation, marital status, religion, employment status, and family support); lifestyle behaviors (smoking and alcohol use); obstetrics or maternal (parity, mode of pregnancy delivery, antepartum hemorrhage); and anthropometric (pregravid body mass index) characteristics of participants. The latter part of the questionnaire solicits participant's information on the perceived barriers to prenatal physical activity and exercise. The items on the perceived barriers to prenatal physical activity and exercise were self-developed based on extensive review of the literature (7, 11, 13, 15, 16, 21, 22, 33). We defined barriers as anything hindering one from engaging in physical activity or exercise (34).

### Qualitative Data

The interviews were conducted in English and lasted between 45 and 60 min in a quiet health facility room to ensure a non-threatening, comfortable and free environment, which encouraged freedom of speech. Participants gave consent to audio-record their interviews, which were transcribed verbatim. The participants expressed their views freely on the barriers to prenatal physical activity. To ensure accuracy and validity, the transcripts were cross-checked with the audio-recorded interviews, and similarly, participants were provided with the interview transcripts and emergent themes for comment and confirmation regarding the accuracy of the interviews.

### Data Analysis

**Figure 1:**
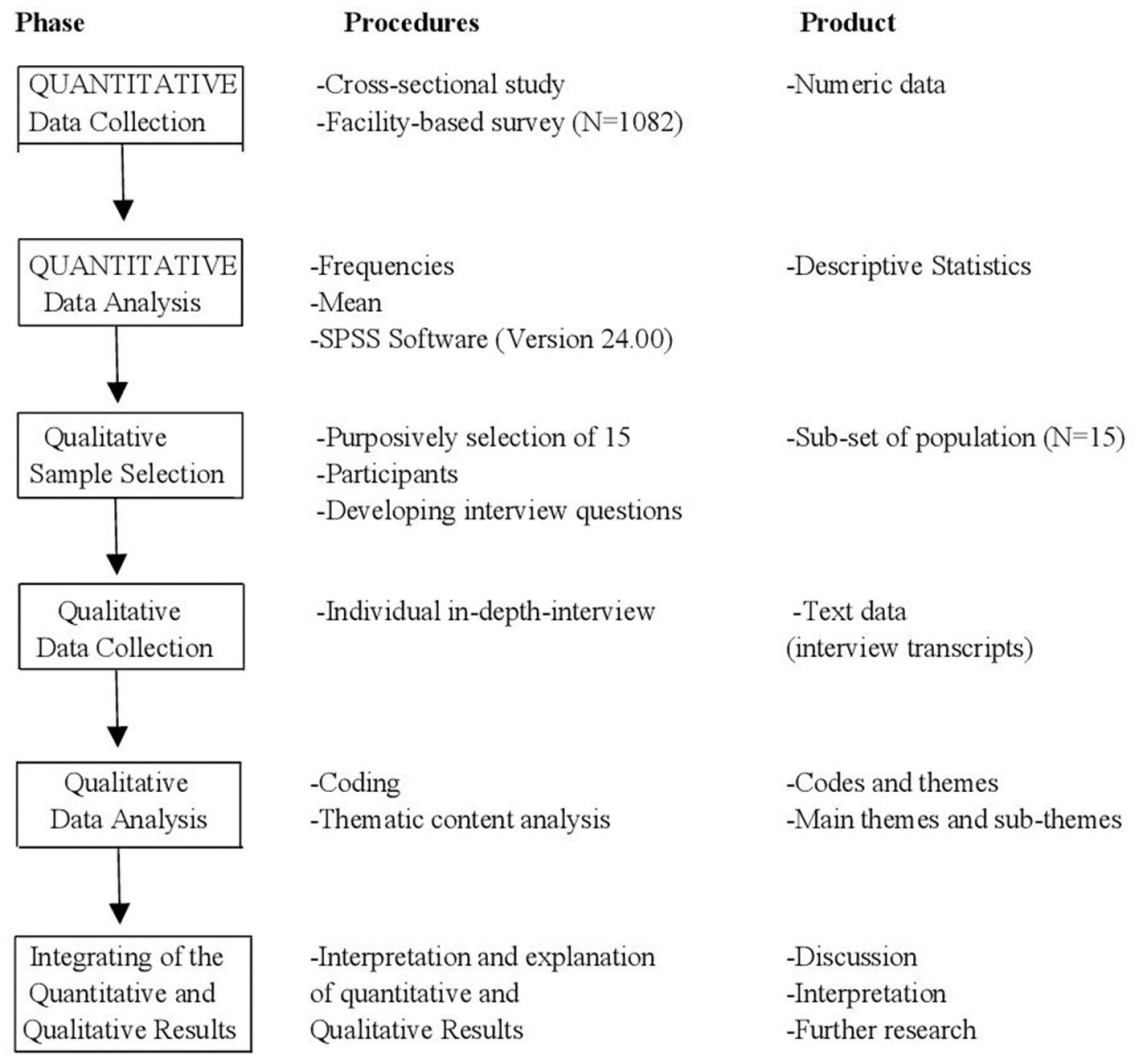
Chart flow of the mixed methods of the study.

We analyzed the quantitative and qualitative datasets separately. Descriptive statistics of mean, frequency and percentages were used to analyse the quantitative data using the Statistical Package for Social Sciences (SPSS) (Version 24.0, IBM SPSS, Chicago, IL, USA); while the in-depth semi-structured interviews were analyzed using the thematic content analysis method, following Tesch's procedure for qualitative analysis (35). An independent coder analyzed the transcripts and identified emerging themes. Applying the principles of explanatory sequential mixed methods study design (26), both the quantitative and qualitative results were merged and interpreted as a comprehensive summary of the total findings (26). Shown in Figure 1 is the mixed methods design flow chart.

## Results

### Quantitative

The participants' socio-demographic, obstetrics and lifestyle characteristics have been described elsewhere (32, 36). Briefly, most of the participants were of black race (86.4%), had never married (66.3%), and had a secondary education (74.2%). Additionally, 67.7% were unemployed, had not experienced antepartum hemorrhage (93.6%), and were having both a vaginal and Caesarian (49.7%) delivery. The majority of the participants had a normal pregnancy body mass index (18.5–24.9 kg/m^2^) (84.8%), nearly two-thirds received no physical activity advice (64.7%), had not participated in pre-pregnancy physical activity (65.1%), and had failed to participate in physical activity during pregnancy (69.6%).

Shown in Table 1 are the barriers to physical activity and exercise. The major barriers to physical activity and exercise cited by the women were inadequate information from healthcare professionals (85.3%), feeling of tiredness (73.3%), lack of advice from healthcare professionals (nurses/midwives) (64.7%), and low energy (64.5%). Other barriers included insufficient and contradictory information (64.8%), non-accessibility to physical activity facilities (63.0%), lack of transport to the gym for exercise (82.3%) and the money to pay for the gym fee (80.1%).

**Table 1:** Barriers to physical activity and exercise (*n* = 1,082).

**Barriers**	**Response distribution**			
	**SA** ***n* (%)**	**A** ***n* (%)**	**D** ***n* (%)**	**SD** ***n* (%)**
Feeling of tiredness	194 (17.9)	599 (55.4)	212 (19.6)	77 (7.1)
Low energy	175 (16.2)	523 (48.3)	329 (30.4)	55 (5.1)
Feeling of illness and morning sickness	86 (8.0)	261 (24.1)	635 (58.7)	100 (9.2)
Feel nausea, vomiting and back pain	97 (9.0)	257 (23.7)	622 (57.5)	106 (9.8)
Feeling of discomfort	74 (6.8)	473 (43.7)	417 (38.6)	118 (10.9)
No one to exercise with	64 (5.9)	499 (46.1)	399 (36.9)	120 (11.1)
Advised to avoid exercise	86 (8.0)	255 (23.6)	640 (59.1)	101 (9.3)
Lack of support from family or friends	45 (4.2)	322 (29.8)	616 (56.9)	99 (9.1)
Partner and family dislike my involvement in physical activity	28 (2.6)	172 (15.9)	766 (70.8)	116 (10.7)
Conflicting advice about physical activity and exercise	88 (8.1)	579 (53.5)	308 (28.5)	107 (9.9)
Cultural dislike or disapproval	12 (1.1)	112(10.4)	811 (74.9)	147 (13.6)
Work commitment	83 (7.7)	253 (23.4)	529 (48.9)	217 (20.1)
Childcare and responsibilities	89 (8.2)	275 (25.4)	573 (53.0)	145 (13.4)
Lack of transport to go to the gym	391 (36.1)	500 (46.2)	120 (11.1)	71 (6.6)
Lack of recreational facilities in the area	130(12.0)	457 (42.3)	408 (37.7)	87 (8.0)
Lack of money to pay for gym fee	273 (25.2)	594 (54.9)	145 (13.4)	70 (6.5)
Lack of access to physical activity facilities	204 (19.0)	476 (44.0)	345 (31.7)	57 (5.3)
Environment not safe to exercise	54 (5.0)	351 (32.4)	572 (52.9)	105 (9.7)
Weather conditions	52 (4.8)	380 (35.1)	183(16.9)	467 (43.2)
Lack of advice and support on the benefits of physical activity	129 (11.9)	568 (52.5)	325 (30.0)	60 (5.6)
Insufficient and contradictory information	237 (22.0)	463 (42.8)	272 (25.0)	110 (10.2)
Lack of advice from healthcare professionals	218 (20.2)	482 (44.5)	268 (24.8)	114 (10.5)
Lack of clear advice about the intensity and dose of exercise	322 (29.8)	601 (55.5)	130 (12.0)	29 (2.7)
Large body weight	37 (3.4)	141 (13.0)	739 (68.3)	165 (15.3)

*SA, Strongly agree; A, Agree; D, Disagree; SD, Strongly disagree*.

### Qualitative

The mean age of participants interviewed was 29.4 ± 3.2 years (*n* = 15). The characteristics of the participants have been described elsewhere (37). Four main themes regarding the barriers to prenatal physical activity were identified (Table 2).

**Table 2:** Themes and sub-themes reflecting the responses of pregnant women regarding barriers to physical activity during pregnancy.

**Themes**	**Sub-themes**	**Example quotes**
Individual or personal barriers	Physical constraints (fatigue, laziness, dizziness, nausea)	“*My mother and mother in-law sometimes asked me to exercise but it is hard for me to exercise because I am always tired after work.”* “*So, for me, I am lazy to exercise. I like to stay at home rather than to walk around.”* “*Well I think that if the woman feels comfortable to continue physical activity during pregnancy…she can do it. Yhoooo, but me, I can't because I am too lazy to exercise, I just want to sleep.”* “*I am always tired, sister. I rather sit at home and watch television.”* “*I get too tired to do exercise during pregnancy.”* “*I still feel nauseous and vomit non-stop.”* “*In the morning or when I take that medication…you dissolve in water (calcium carbonate). After the vomit, I don't want to do anything.”* “*I still vomit but not always like before, only certain food (smell) still make me vomit or nauseous.”*
	Body exhaustion	“*I usually have low energy to do anything, but I don't vomit and I no longer feel morning sickness.”* “*My area is safe to exercise but I don't have energy.”* “*I usually feel so heavy especially now that I am close to my delivery. I feel like I am carrying heavy load, so after work, when I came back, I feel tired and want to sleep non-stop.”*
	Safety concerns	“*I was not thinking that a pregnant woman should be exercising because for me, what if she gets hurt and ends up hurting her baby?”* “*I thought it's dangerous to the baby, maybe it may cause an abortion or maybe I might bleed or hurt the baby.”* “*Sister, there is something I didn't tell you before, back at home especially we Africans, I was told that it is not good to exercise during pregnancy: one must avoid hurting oneself as well the baby.”*
	Lack of time	“*Another reason why I don't exercise is a lack of time. My classes start by 08:00 and end by 14: 00 every day, so that's why I get so tired all the time. It's like I don't have enough energy to do anything, not even to study my books these days. And when I am not doing anything–especially during weekends–I sit around, I also watch television.”* “*I don't exercise regularly because of the time factor.”*
	Work, school, household and childcare responsibilities	“*I am usually tired after work.”* “*I also feel tired but I must finish preparing food, doing washing and cleaning. Most times I look after my sister's child the whole day, it is tiring.”* “*Well, I don't exercise but I also do a lot of other things, I walk around a lot, and when I get a job, I clean, wash, iron and sometimes cook. I think that is enough for me.”* “*I also feel tired after I come back from school. And by weekends, is the only time I rest and do my laundry and cleaning.”*
Lack of information barriers	Lack of knowledge on the importance of physical activity during pregnancy	“*No Ma'am, they don't really talk about it, except one time the sister said to me that I should walk a lot now that I am pregnant because it is good. Other than that, no they did not tell us about any benefit and how often or the type of physical activity to do during pregnancy.”* “*I was not told about the health benefits of physical activity during pregnancy.”* “*I don't have enough information on exercise during pregnancy.”* “*I don't know that exercise is recommended during pregnancy.”* “*I don't know the type of exercise and the duration of the exercise.”* “*I have no idea that a pregnant woman should exercise during pregnancy.”*
	Lack of prenatal physical activity advice or counseling from midwives	“*I didn't received any information about physical activity and exercise during pregnancy, not even from the nurses.”* “*I have never been told about physical activity during pregnancy by a nurse.”* “*Yhoo!! I am not aware of physical activity during pregnancy nor the type of physical activity to do during pregnancy.”*
Lack of resources barriers	Insufficient finances	“*I usually find it difficult to exercise, not because I don't want to, but the price to register in the gym is costly. I'm not working.”* ***“**Yhooooo!!! Where I stay is very far from the gym, so it is very inconvenient for me.”* “*About gyming, yhooo!!! Gym, noooooo!!! I don't gym, even if I wanted to, I don't have money to register at the gym.”*
Environmental barriers	Weather conditions	“*The weather is so cold, even now that I am talking with you, I am feeling cold.”* “*I can't exercise in this cold weather.”* “*Weather is cold for exercise.”* “*In this weather, yhooo!! It's still cold, sister.”* “*The weather is so cold. I can't exercise in this cold weather.”*
	Safety neighborhood	“*My area is not safe for someone to go out and exercise.”* “*Fear of being attacked by criminals in the street while walking or jogging late evening.”*

### Individual Barriers

Tiredness as a physical constraint was the barrier mentioned most by the participations. The participants expressed feeling tired after work and performing household chores and other commitments. They further expressed that they lacked the time to engage in physical activity or exercise. They narrated thus:

“*I am always tired after work*.”
“*I am lazy to exercise*.”
“*I am always tired, sister; I rather sit at home and watch television*.”

The participants further maintained they feel exhausted and have low energy, that is, not enough to engage in physical activity during pregnancy:

“*I usually have low energy to do anything*.”
“*I usually feel so heavy, especially now that I am close to my delivery. I feel … carrying heavy load …I feel exhausted ….to sleep non-stop*.”

The women felt concern about the safety of physical activity during pregnancy. They indicated that participating in physical activity would hurt the mother and the baby. They narrated:

“*I …. what if she gets hurt and ends up hurting her baby*?”
“*I thought it's dangerous to the baby, maybe it may cause an abortion or maybe I might bleed or hurt the baby*.”
“*I was told it is not good to exercise during pregnancy; one needs to avoid hurting oneself as well the baby*.”

Furthermore, the lack of time was a factor restraining the women from prenatal physical activity. The lack of time was related to their household and work commitments. Some of the women highlighted thus:

“*I'm too busy doing other things in the house. I have not time because I don't have someone to help me. Sisi, time, time is my problem*.”
“*I don't exercise regularly because of time*.”
“*I am usually tired after work*.”
“*I also feel tired after I come back from school. And by weekends, is the only time I rest and do my laundry and cleaning*.”

### Lack of Information

The participants mentioned a lack of knowledge of the importance of physical activity during pregnancy as another barrier to their prenatal physical activity practice. They maintained having no or limited knowledge about prenatal physical activity. In addition, the midwives whom they consult during antenatal healthcare sessions, did not provide advice or counseling about physical activity during pregnancy. They highlighted as follows:

“*I don't have enough information on exercise during pregnancy*.”
“*I don't know the type of exercise and the duration of the exercise*.”
“*I didn't receive any information about physical activity and exercise during pregnancy, not even from the nurses*.”
“*Yhoo!! I am not aware of physical activity during pregnancy, nor the type of physical activity to do during pregnancy*.”

### Lack of Resources

The women expressed that they lacked the resources to afford registration at a gym and could not pay for it. They maintained:

“*The price to register at the gym is high. I cannot afford the price. I'm not working*.”
**“***Yhooooo!!! Where I stay is very far from the gym, so it is very inconvenient for me*.”
“*I don't gym; even if I wanted to, I don't have money to register at the gym*.”

### Environmental Barriers

Most of the participants indicated that generally, their areas are not safe to go out for physical activity or exercise. The cold weather do sometimes constitute a barrier to their prenatal physical activity or exercise practice. In addition, the fear of being attacked by some criminal individuals in the street is possible. They narrated thus:

“*My area is not safe for someone to go out and exercise because of fear of being attacked by criminals in the streets, especially if it is late in the evening.”*
“*It is sometimes very cold in the winter season to go out for walking or jogging in the street*.”
“*Sometimes the weather is too cold, as you can feel it now. I can't exercise in this cold weather*.”
“*I feel the weather is too cold to go out for jogging or walking*.”

## Discussion

This is the first study to use a mixed-methods approach to assess the barriers to prenatal physical activity among pregnant women in the Eastern Cape Province, in South Africa. Understanding the reasons restraining pregnant women from being physically active during pregnancy may provide useful information on how to design context-specific interventions to address the factors in order to promote prenatal physical activity in this region. The present study revealed that inadequate information from healthcare professionals about the intensity and dose of exercise, lack of advice from healthcare professionals (nurses/midwives), insufficient and contradictory information about prenatal physical activity, and conflicting advice about physical activity and exercise hinders women from practicing prenatal physical activity. We confirmed the above findings in the qualitative findings. The participants repeatedly mentioned that the midwives do not offer advice on prenatal physical activity. Our results thus align with findings from other qualitative studies which reported women receiving no or minimal and ineffective physical activity counseling (1, 18). As maintained by Sytsma et al. (7) effective counseling of pregnant women entails the need to identify and understand the barriers to prenatal physical activity; furthermore, it was found that women who received physical activity advice or counseling from healthcare providers are more likely to practice and maintain their physical activity levels (33, 38, 39). Notably, health care providers are faced with challenges in providing effective prenatal physical counseling, which include a lack of adequate education/training, limited resources and time to address issues pertaining to prenatal physical activity with the women during antenatal consultations (39–41). Nevertheless, information from healthcare providers is very important to pregnant women seeking answers to questions about their maternal health; therefore, addressing their individuals concerns and needs based on scientific evidence would empower women to take informed decisions or choices regarding their maternal health, and in this regard, prenatal physical activity.

Our findings in the quantitative phase of this study showed that tiredness, exhaustion, discomfort, and low energy are the barriers to their physical activity participation. The findings from qualitative findings mirrored the quantitative results. Tiredness, fatigue, work commitments, lack of time have been widely reported in the literature as barriers to physical activity participation during pregnancy (7, 9, 13, 16–18, 22, 33). The participants in the current study repeatedly mentioned that tiredness linked to work and household responsibilities, was a predominate barrier to their physical activity participation; exacerbated by lack of time and feelings of exhaustion drastically limiting their exercise or initiation of any physical activity. As quoted, “*I am always tired after work*” and “*I don't exercise regularly because of time*” spoke to this. In addition, the participants echoed having low energy to engage in physical activity during pregnancy. In other qualitative studies of black South African pregnant women, pregnancy-related discomforts and a lack of time were the perceived barriers to physical activity (1, 5); other studies echoed similar findings (7, 8, 10). Although the physiological changes affecting women- especially in early pregnancy such as nausea, morning sickness and vomiting, were rarely reported among women in this present study, both in the quantitative and qualitative data. A previous study have shown that these physical physiological variables affect the level of prenatal physical activity and exercise (7).

Other barriers mentioned by the women in this present study included the non-accessibility to physical activity facilities and lack of recreational facilities. Pregnant women without access to some basic equipment, facilities, or instructor guidance may limit their participation in physical activity (9, 42–44). Provision of access to appropriate and affordable exercise facilities in the community may aid in facilitating the promotion of physical activity during pregnancy (45, 46).

Similar to other studies (1, 24, 47, 48), women in this current study reported a lack of financial resources to register and pay for gym exercise as a barrier for prenatal physical practice, a finding, stressed in the qualitative finding. “*The price to register at the gym is high. I cannot afford the price. I'm not working*.” Maternal employment is associated with prenatal physical activity practice as women who are unemployed tend to have lower prenatal physical activity (32).

It has been demonstrated that physical activity is safe for both the mother and fetus; it is associated with minimal or rare adverse health complications and is associated with the likelihood of reduced preterm birth, low birth weight, fewer miscarriage and perinatal mortality (33, 48–51). The present study demonstrated that pregnant women felt concern about their safety and the baby during physical activity in pregnancy. This resonated more prominently during the qualitative interviews in which women expressed concern that participating in physical activity may harm the baby. For example, “*I … what if she gets hurt and ends up hurting her baby*?” In studies reporting both quantitative (16, 52) and qualitative (11, 17, 18) findings, women expressed the fear of hurting the baby during physical activity in pregnancy. Our study echoed the findings of Muzigaba et al. (5) whose study indicated most pregnant women were unsure of the safety of exercising during pregnancy and unaware of what type of exercise or physical activity is safely recommended (5). Importantly, in the absence of any contraindications, pregnant women should be encouraged to engage in physical activity; and follow advice regarding prenatal physical activity provided to them regarding the benefits of physical activity in pregnancy (48). This, again, stressed the need for providing prenatal counseling to change the perception and behavior of women toward physical activity in order to improve their physical activity levels during pregnancy.

The findings of this study revealed participating in outdoor physical activity or exercise, for example, jogging and walking is not safe, and the weather constitute a barrier to physical activity or exercise during pregnancy. One-third of the participants perceived the neighborhood environment (37.4%) and cold weather (39.9%) as barriers. This was certainly supported during the individual interview conversations. The participants indicated that the fear of being attacked by some criminal individuals in the street was possible. “*It is sometimes very cold in winter season*.” Women avoided exercising in the neighborhood streets and public open spaces because of a perceived fear of personal safety (47, 53, 54). Seemingly, the women feel more vulnerable to street crime compared to their male counterparts. The fear of unleashed dogs is also possible. However, these variables were not investigated. The findings underscored the need for safe neighborhood and public space interventions for prenatal physical activity. Neighborhood safety may affect the practice of outdoor physical activities, and the utilization of public physical activity facilities such as parks, sports clubs, fitness centers and gyms (55). According to Uddin et al. (56) improving neighborhood safety requires a multidisciplinary approach involving the government provision of infrastructure, local policing and the participation of the local community. Notably, this present study was conducted in a semi urban area; therefore, the issue of personal safety as a barrier seems inconsequential. Although weather was not mentioned as a major barrier to physical activity among pregnant women in our study, other studies cited weather condition as a top deterrent to physical activity (9, 11, 47). Contrastingly, a previous study conducted in the USA, cited weather as a lesser barrier to physical activity (53). The issue of weather is seasonal and varies in different geographical settings. Notwithstanding this connotation, interventions to encourage women to exercise indoors, when the weather conditions are inclement, are desirable. The public physical activity and recreational places should be provided with adequate security and protection from rain and cold; this could help to motivate pregnant women to engage in regular physical activities.

### Implications

This study presents several barriers to prenatal physical activity practice among women in this setting. The findings of this present study might be relevant in different contexts: public health, policy health advisors, and health practitioners concerned with prenatal health of the women. Pointedly, the several barriers reported herein, and in the literature, suggest the need for information sharing and education. These findings from both the quantitative and qualitative approaches regarding the barriers to physical activity during pregnancy among pregnant women in this present study present some salient suggestions for developing context-specific interventions to address the barriers reported by the women in this setting. Seemingly, possible interventions in this regard may include providing advocacy on prenatal physical activity and exercise at the health facilities, where women attend antenatal healthcare; this could be extended at the community level to involve a multidisciplinary healthcare team concerned with maternal health; it could also include printed matter and social media. In addition, we advocate for the need to infuse prenatal physical activity into the curriculum of medical and healthcare professions and the training of healthcare providers on prenatal physical activity prescription, recommendations and guidelines. In turn, the healthcare providers would be better equipped to offer physical activity advice based on scientific evidence during prenatal care. Prenatal care presents a good opportunity to counsel women to achieve an active lifestyle for improved perinatal health outcomes (55). The advocacy dictum “Exercise is Medicine” should be imbibed by healthcare providers to improve the uptake of physical activity during pregnancy; and interventions should focus on individuals' unique needs to be effective. Furthermore, it should be a priority for city policymakers to create recreational facilities for physical activities that women could engage in outside the home setting.

### Limitations

The limitations of the study are worth noting. First, we used a self-reported questionnaire to assess barriers, which may be subject to bias and individual interpretation. In addition, interviews were conducted in English, which might not be the preferred language of black women from Eastern Cape. Secondly, we investigated an important health behavior for pregnant women, in a diverse, understudied and predominantly black population; however, the study made use of a sample of women seeking only antenatal healthcare in public health facilities in Buffalo City Municipality in the Eastern Cape Province. Therefore, the findings of this study cannot be generalized to pregnant women attending private health facilities nor to other regions of the Eastern Cape or provinces in South Africa. In addition, we qualitatively explored the barriers to physical activity with a relatively small percentage of the women under study, which further limits generalization of the results of the study. However, the primary focus of conducting qualitative study is to gain insights on a matter, rather than to generalize the findings.

Future studies in other settings of South Africa are needed to provide a wider context of the constraining factors for prenatal physical activity of women to inform wide-scale governmental and community-based interventions to address the barriers. In addition, longitudinal studies are needed to ascertain the barriers to physical activity pre, during and after pregnancy, as ours was a cross-sectional study. It is also important to determine which prenatal physical activity interventions are most effective over time.

## Conclusion

The findings of this study highlight a range of constraining factors that hinder pregnant women to engage in prenatal physical activity. Majorly, women reported tiredness, lack of time, exhaustion, discomfort, and low energy. In addition, they mentioned lack of advice, information and support about prenatal physical activity. These findings calls for a context-specific interventions to address the barriers to prenatal physical activity in this setting.

## Data Availability Statement

The original contributions presented in the study are included in the article/supplementary material, further inquiries can be directed to the corresponding author.

## Ethics Statement

The study protocol was approved by the University of Fort Hare's Health Research Ethics Committee. The patients/participants provided their written informed consent to participate in this study.

## Author Contributions

UO and DG: conceptualization and drafting of manuscript. UO: data collection. DG: review and editing and supervision. All authors read and approved the final manuscript.

## Funding

The work reported herein was made possible through funding by the South African Medical Research Council through its Division of Research Capacity Development under the Bongani Mayosi National Health Scholars Programme from funding received from the Public Health Enhancement Fund/South African National Department of Health.

## Author Disclaimer

The content hereof is the sole responsibility of the authors and does not necessarily represent the official views of the SAMRC.

## Conflict of Interest

The authors declare that the research was conducted in the absence of any commercial or financial relationships that could be construed as a potential conflict of interest.

## Publisher's Note

All claims expressed in this article are solely those of the authors and do not necessarily represent those of their affiliated organizations, or those of the publisher, the editors and the reviewers. Any product that may be evaluated in this article, or claim that may be made by its manufacturer, is not guaranteed or endorsed by the publisher.
